# Characterization of a B16-F10 melanoma model locally implanted into the ear pinnae of C57BL/6 mice

**DOI:** 10.1371/journal.pone.0206693

**Published:** 2018-11-05

**Authors:** Marine Potez, Verdiana Trappetti, Audrey Bouchet, Cristian Fernandez-Palomo, Esra Güç, Witold W. Kilarski, Ruslan Hlushchuk, Jean Laissue, Valentin Djonov

**Affiliations:** 1 Institute of Anatomy, University of Bern, Bern, Switzerland; 2 Institute of Bioengineering and Swiss Institute for Experimental Cancer Research, École Polytechnique Fédérale de Lausanne, Lausanne, Switzerland; Istituto Superiore di Sanità, ITALY

## Abstract

The common experimental use of B16-F10 melanoma cells focuses on exploring their metastatic potential following intravenous injection into mice. In this study, B16-F10 cells are used to develop a primary tumor model by implanting them directly into the ears of C57BL/6J mice. The model represents a reproducible and easily traceable tool for local tumor growth and for making additional *in vivo* observations, due to the localization of the tumors. This model is relatively simple and involves (i) surgical opening of the ear skin, (ii) removal of a square-piece of cartilage followed by (iii) the implantation of tumor cells with fibrin gel. The remodeling of the fibrin gel within the cartilage chamber, accompanying tumor proliferation, results in the formation of blood vessels, lymphatics and tissue matrix that can be readily distinguished from the pre-existing skin structures. Moreover, this method avoids the injection-enforced artificial spread of cells into the pre-existing lymphatic vessels. The tumors have a highly reproducible exponential growth pattern with a tumor doubling time of around 1.8 days, reaching an average volume of 85mm^3^ 16 days after implantation. The melanomas are densely cellular with proliferative indices of between 60 and 80%. The induced angiogenesis and lymphangiogenesis resulted in the development of well-vascularized tumors. Different populations of immunologically active cells were also present in the tumor; the population of macrophages decreases with time while the population of T cells remained quasi constant. The B16-F10 tumors in the ear frequently metastasized to the cervical lymph nodes, reaching an incidence of 75% by day 16. This newly introduced B16-F10 melanoma model in the ear is a powerful tool that provides a new opportunity to study the local tumor growth and metastasis, the associated angiogenesis, lymphangiogenesis and tumor immune responses. It could potentially be used to test different treatment strategies.

## Introduction

The incidence of melanoma is increasing worldwide. The annual occurrence rates for new melanoma cases per 100,000 inhabitants are by 10–25 patients in Europe, 20–30 in the U.S.A. and 50–60 in Australia [1].

To date, systemic therapies, including chemo-, immuno- and targeted therapy, are the most studied and most promising treatment alternatives. A proper understanding of local tumor growth and invasion mechanisms is particularly important to advance the treatments for melanoma [1]. *In vivo* studies are crucial to monitor the early local events such as tumor onset, expansion and invasion of adjacent tissue, local inflammatory and immune response, blood and lymphatic vessel development and metastasis propagation, events that are difficult to evaluate *in vitro*. Elucidating these mechanisms is essential for the creation of new and more effective treatment strategies.

Many melanoma models have been developed, such as xenografts (from human tumors) [2], syngeneic models (genetically identical tumors) [3–5], induced tumors (with carcinogen or UV exposition) [6,7], or the so-called “genetically engineered models” (with an overexpression of oncogenes or inactivation of tumor suppressor genes) [8–11].

Transplanted syngeneic models have been used with different types of melanoma such as the Cloudmans91 melanoma in DBA/2 mice [4], the Harding-Passey melanoma in BALB/c x DBA/2F1 mice [3] or the B16 melanoma in C57BL/6 mice [5]. In more detail, different B16 melanoma sublines have commonly been used, namely: the B16-F1, a non-selected cell line with low potential to metastasize; the B16-F10, selected for its capacity, *in vivo*, to produce distant metastases; and the B16-BL6, selected for its highly invasive capacity *in vivo* [12]. Depending on the study and the model, a method of tumor induction will be selected. For example, the B16-F10 cells can be injected via the tail vein to produce artificial metastases, mainly in the lung. The B16-BL6 cells can also be injected in the footpad to observe the development of metastases after amputation of the foot [13]. Finally, the chosen cells can be injected into the skin of the flank [14] or the hind leg [15,16] to study local tumor growth or spontaneous metastatic development. Other studies have used the subcutaneous injection of melanoma cells into the ear [17,18]. However, the intradermal injection of tumor cells, although fast, has the disadvantage of increasing intradermal pressure which forces tumor cell entry into the lymphatic vessels and lymph nodes [19]. The use of the locally injected model is therefore inappropriate for the study of the process of spontaneous lymph node metastasis.

In the present study a detailed characterization of the B16-F10 melanoma cell line as a model of a primary tumor in the ear of C57BL/6 mice, is presented along with a description of (i) the implantation procedure and the kinetics of tumor growth; (ii) blood and lymphatic vascularization; (iii) the tumor and immune cell features; and (iv) the spontaneous development of metastases.

## Material and methods

### Animals

All the animal experiments were conducted after the approval by the veterinary office of the Canton of Bern under permit BE61/15. C57BL/6J mice (females, 8 weeks old, Charles River Laboratories France), weighing between 18 g and 22 g, were used in this study. The mice were housed in standard cages at 21°C with a 12h light- 12h dark cycle and had access to food (standard laboratory chow) and water *ad libitum*. For experimental procedures, mice were anesthetized by an intraperitoneal injection (i.p.) of a mixture of fentanyl (0.05 mg/kg body weight (bw)), midazolam (5 mg/kg bw) and medetomidine (0.5 mg/kg bw), with 5.6 ml/kg bw of a 0.9% sodium chloride solution.

The behavior of the mice (when moving, feeding or interacting with other mice) was judged to be normal during the whole period of the study. All mice were killed humanely by an i.p. injection of pentobarbital (50 mg/kg bw) at the end of the study (16 days) or earlier for sample collection or if the tumor ulcerated.

### Cells

The B16-F10 melanoma cells were a gift from Ecole Polytechnique Fédérale de Lausanne (EPFL), but originally obtained from the American Type Culture Collection (ATCC). The cells were cultured in complete Dulbecco's Modified Eagle's Medium (DMEM high glucose, Gibco) supplemented with 10% (v/v) Fetal Bovine Serum (FBS, Biochrom) and 2% (v/v) Antibiotic-Antimycotic (Gibco), and maintained at 37°C in a humidified incubator with 5% CO_2_.

### Tumor implantation

The surgical procedure has been adapted from that of Güç *et al*. [19]. Tumor cells were implanted in a mixture of thrombin and fibrinogen to create a new matrix as briefly outlined below.

**Fig 1:**
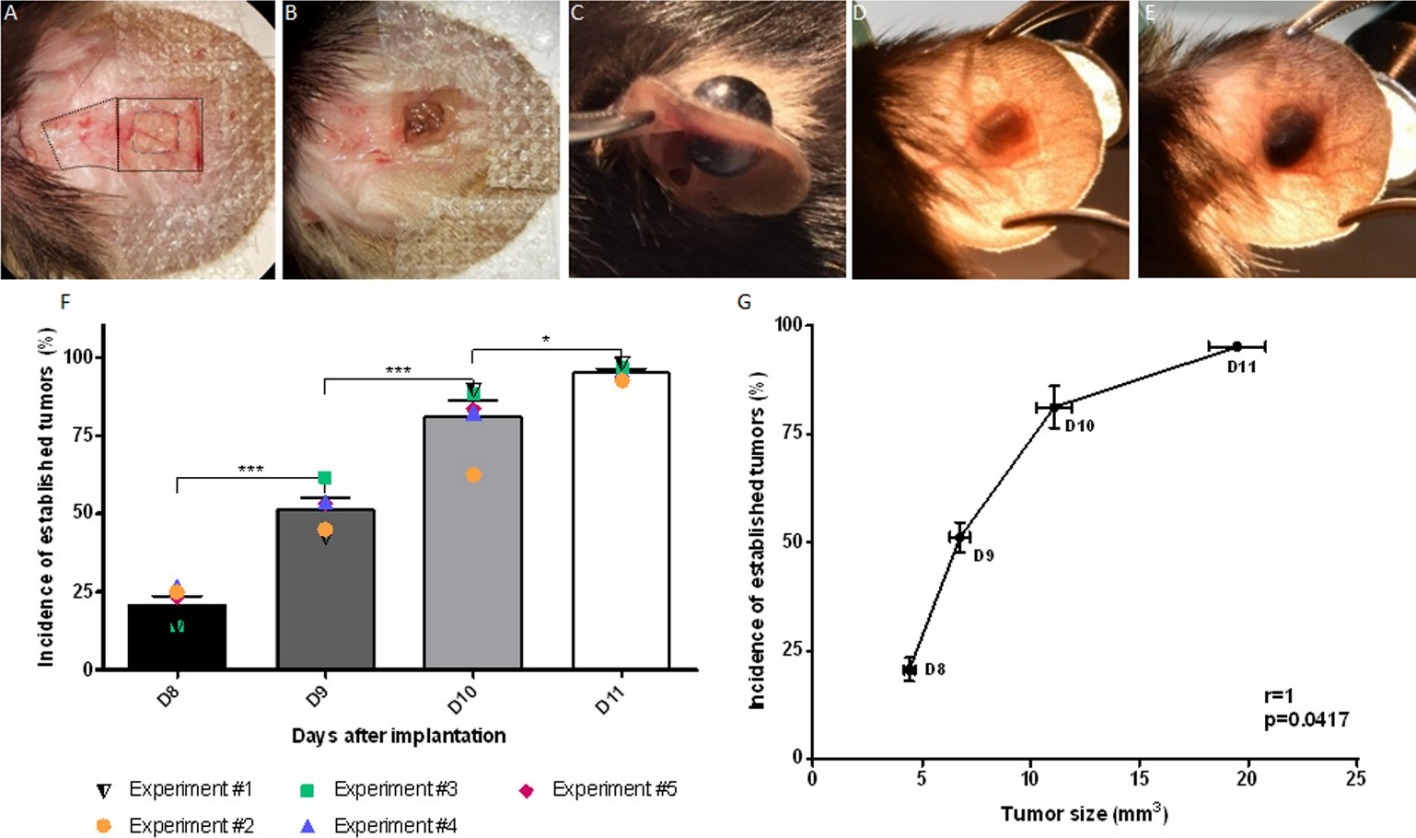
Tumor inoculation method. (A-B) Ventral view of an ear during surgery (A: dotted square = area from ventral skin flap reflected; small square = outline of the region of full thickness cartilage to be removed (4 mm^2^). Clot containing tumor cells (B: brown nodule) placed on the region from which cartilage removed. (C) A large black tumor growing on both sides of the ear (day 13). (D) Dorsal view of an ear showing a non-established tumor (greyish) (day 8). (E) Dorsal view of an ear established black tumor (day 11). (F) Time related changes in the mean percentage of “established tumors” as evaluated by color. The mean percentage of black tumors detected between day 8 and 11 based on the 5 different studies are represented by the error bars. Results for each individual study are represented by colored symbols. (G) Correlation plot between the percentage incidence of black tumors and their size as a function of time after trans-plantation. r: correlation coefficient. Data shown as mean ± SEM. *: p<0.05, ***: p<0.001, ****: p<0.0001.

After induction of anesthesia the mouse was placed on its back and the ear was fixed on a a stack of glass slides by 3 pieces of adhesive tape (S1A Fig). Three scalpel incisions were made in the skin, each 5 mm long, centered in the ventral side of the ear as measured between edges of the ear and its anti-helix and done at the anterior, posterior and distal end of a projected square (S1A Fig). The skin was then peeled back, in the form of a skin flap, to expose the underlying cartilage (S1B Fig). Four shorter incisions were made in the cartilage (S1C Fig) and the smaller square (roughly 4 mm^2^) of cartilage was removed with forceps (Fig 1A and S1D Fig). A clotted extemporaneous mix of 3 μl of thrombin solution (18 U/ml with 2 mM CaCl_2_, Sigma) and 3 μl of fibrinogen (3 mg/ml, Sigma), containing 4 μl of B16-F10 cells (120’000 cells) was used to fill the opened cavity created (Fig 1B and S1E Fig). The opened ear was closed, by returning the skin flap to its original position (while keeping the implanted clot, containing the tumor cells in the center). The edges were re-attached with surgical glue (Histoacryl L, Braun) (S1F Fig). Tumors started to become visible six to eight days after implantation (S2A to S2D Figs). They grew in all directions following the shape of a sphere (Fig 1C).

### Criteria for established tumors

The establishment of the tumors was evaluated by their color (becoming black) from 8 to 11 days post-implantation (dpi). Initially, the cartilage-free area of the ear was translucent but became black as the tumor grew. The percentage of black tumors (Fig 1E), compared to the total number of tumors implanted, is shown in Fig 1F. The data were obtained from 5 replicate experiments with a total of 470 tumors. All animals showing a non-black mass on day 11 (D11, 4.8%) were deemed as a failure of both the surgery and the tumor growth; and therefore were excluded from the study.

### Growth curves

**Fig 2:**
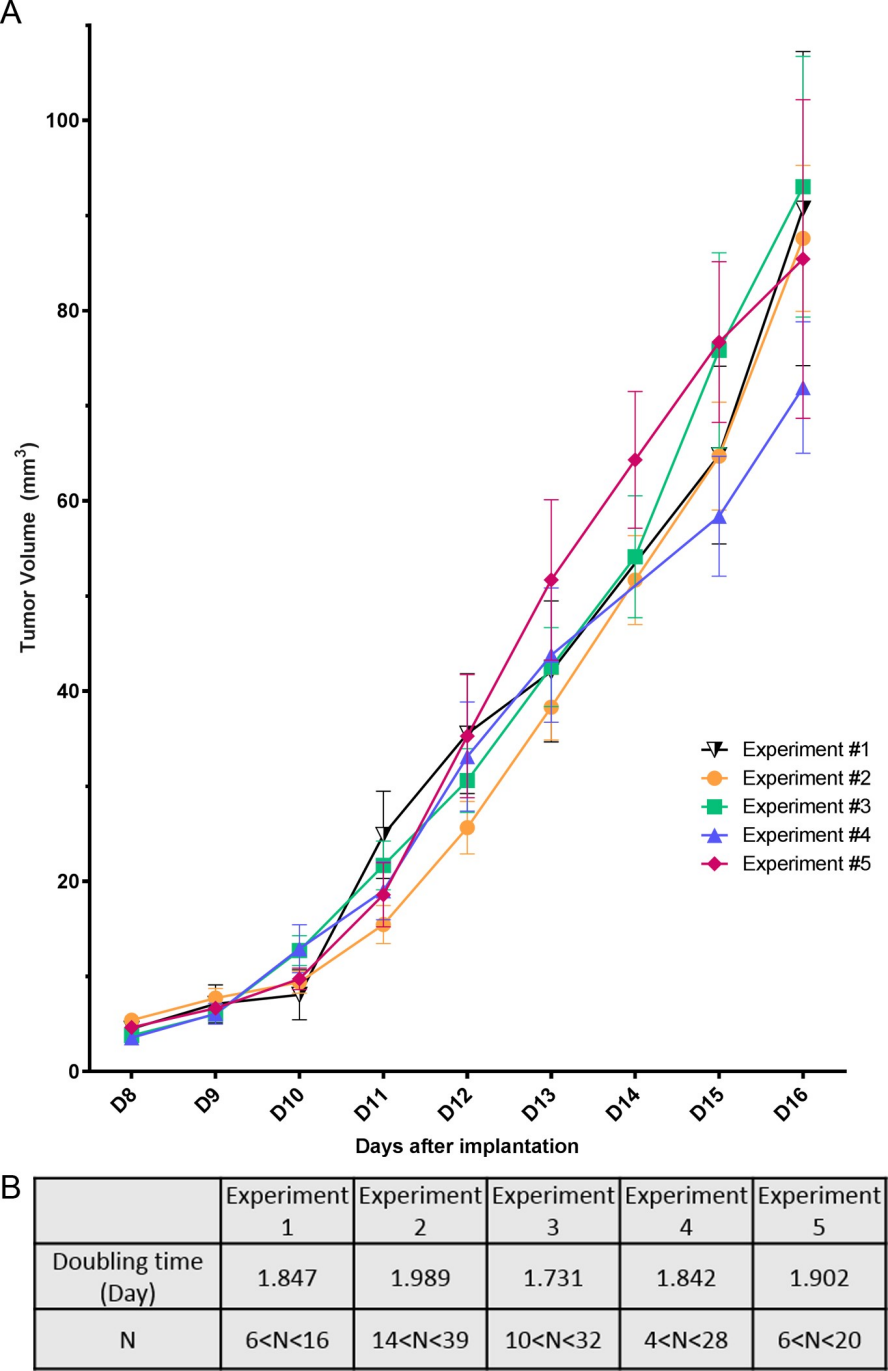
Tumor growth and reproducibility. (A) Changes in tumor volume as a function of time after implantation. Tumor growth curves are shown for the 5 different studies (each individual experiment is represented by a colored symbol). Data shown as mean ± SEM. (B) Tumor doubling times and the number of tumors (N) available for measurements, per repeat study, at day 8 (the highest number) the lowest number day 16.

The tumors growth curves (Fig 2A), were created from 5 replicated experiments, with a total of 135 tumors. The distribution of animals within each replicate experiment is given in Fig 2B, with N = number of tumors; the highest number = number of tumor on D8; the lowest number = number of tumors on D16. The non-constant number of tumors is due to sample collection, at different time points, or ulceration.

The data were obtained by performing daily measurements of the tumor’s size by registering the length of their x, y, and z axis of the tumor with a digital caliper. The tumor volume (mm^3^) was calculated with the formula:

$$V\;=\;\frac{4}{3}\pi \;\times \;\frac{a}{2}\;\times \;\frac{b}{2}\;\times \;\frac{c}{2}$$

where a, b and c are the respective lengths of the tumor on the x, y and z axis. The tumor on each mouse was measured daily, starting at D8 after implantation. The tumor doubling times were calculated from D8 to D16.

### Immunohistochemistry and quantification

At post-mortem, each tumor was frozen in liquid isopentane and then stored at -80°C. The tumors were cut transversally (frozen sections of 12μm thick) in the latero-medial direction.

**Fig 3:**
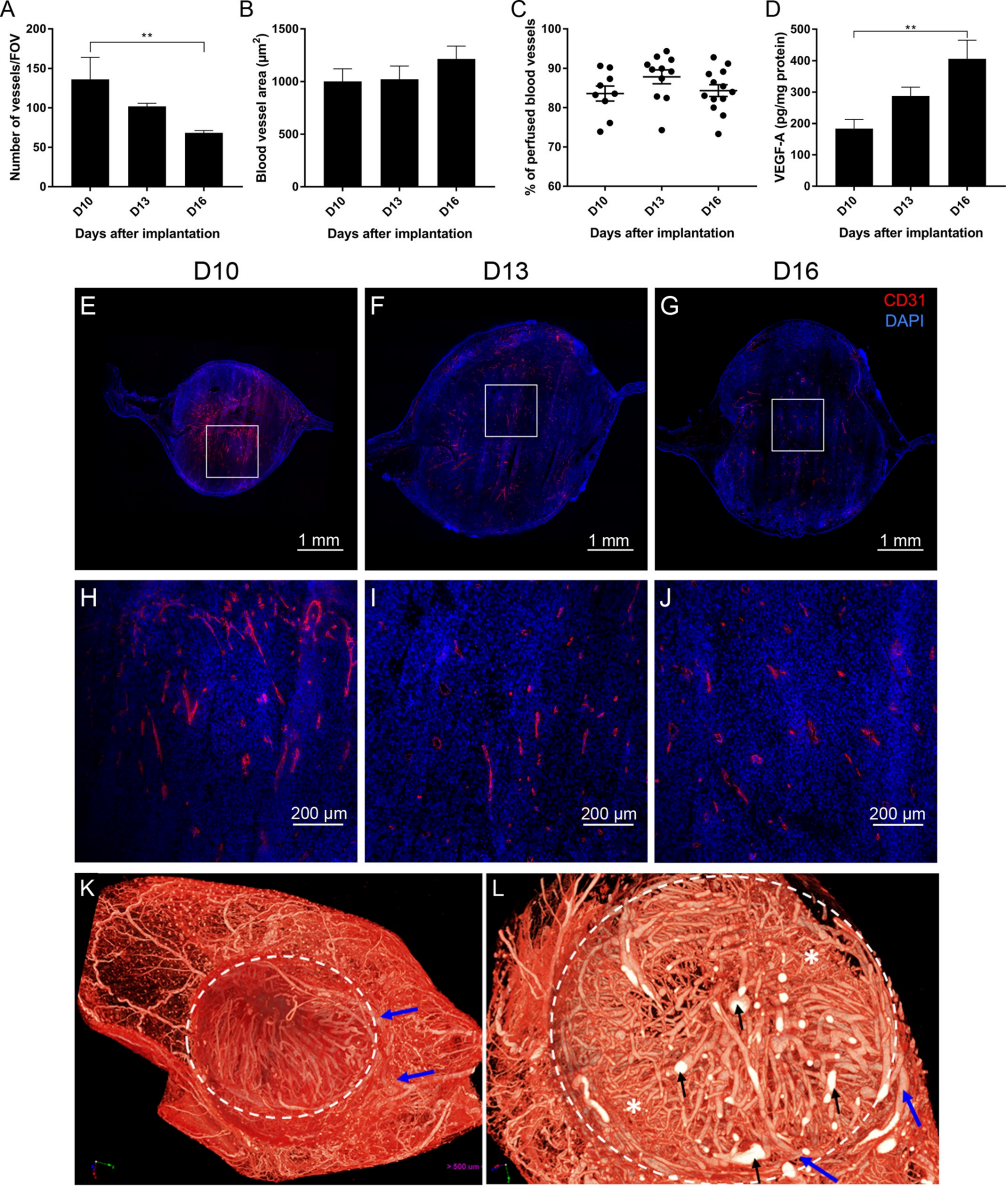
Hemic tumor vascularization: Vascular density, vascular area and perfusion. (A) Time related changes in the number of blood vessels per field of view on days 10, 13 and 16 after implantation. (B) Time related changes in the blood vessel surface area (in μm^2^) at 10, 13 and 16 days after implantation. (C) Time related changes in the percentages of perfused blood vessels. Each symbol represents the value for a single tumor, the horizontal lines mean percentage. (D) Concentration of VEGF-α produced in tumors at 10, 13 and 16 days after implantation. (E, F, G) Representative merged mosaics of CD31 and DAPI staining showing the blood vessels in tumors at D10 (E), D13 (F) and D16 (G) after implantation. White squares: Areas enlarged in Fig 3G, 3H and 3I. (H, I, J) Zoom *in* of pictures D (H), E (I) and F (J), represented by the white square representing the vascular density at higher magnification. (K, L) MicroangioCT of a melanoma on D13. (K) 3D reconstruction of the whole mouse ear and its vasculature including the implanted tumor (encircled). The main feeding vessels are coming from the base of the ear (blue arrows). (L) Virtual section through the tumor at higher magnification (= 3D-microangioCT dataset). A feeding vessel (blue arrow) is entering the tumor mass. Areas with dense network of smaller vessels (asterisks) and many bigger vessels (black arrows). Data shown as mean ± SEM. **: p<0.01. Scale bars: 1 mm (E, F, G) and 200 μm (H, I, J).

**Fig 4:**
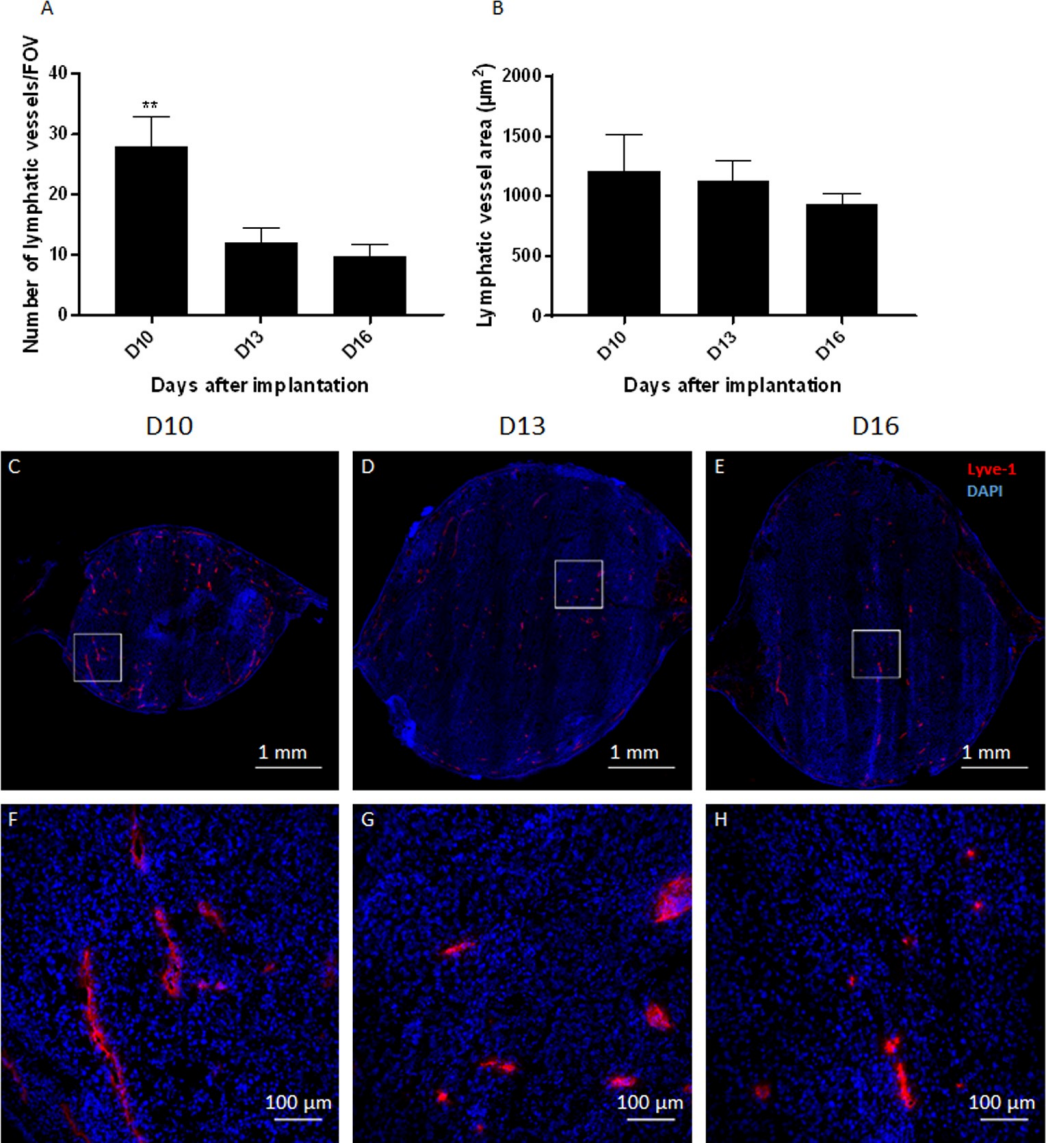
Lymphatic tumor vascularization: Density and area. (A) Time related changes in the number of lymphatic vessels per field of view. (B) Time related changes in lymphatic vessel surface area per field of view (in μm^2^). (C, D, E) Representative merged mosaics of Lyve-1 (red) and DAPI (blue) staining showing lymphatic vessels in tumors on D10 (C), D13 (D) and D16 (E) after implantation. (F, G, H) Zoom *in* of white square in pictures D (F), E (G) and F (H) represented tumor lymphatics at higher magnification. Data shown as mean ± SEM. **: p<0.01. Scale bars: 1 mm (D, E, F) and 100 μm (G, H, I).

To evaluate the presence of lymphatic vessels (Lyve-1, ReliaTech) and the fraction of proliferating cells (Ki67, Thermo Fisher Scientific), the slices of six to seven tumors per time point were fixed with paraformaldehyde (PFA) for 10 min and blocked with donkey serum diluted in in phosphate buffered saline (PBS, 5% v/v) for 30 min. The slices were incubated with the primary antibodies (Lyve-1 1/200, Ki67 1/500) overnight at 4°C, followed by the secondary antibodies (Alexa 488 or Alexa 568, Thermo Fisher Scientific) for 2h at room temperature. The slices were finally washed with PBS containing 4',6-diamidino-2-phenylindole (DAPI, 5 μg/ml, Invitrogen) and mounted using fluoromount (Sigma). Images were taken using a Zeiss Axioplan2 microscope with Visiview software (objective x10 for Lyve-1, field of view (FOV) 1.864 mm^2^, and x20 for Ki67, FOV 0.466 mm^2^). The CD31 (BD Pharminogen) and Lyve-1 mosaics were performed on a Leica M205FA microscope with the Leica software (LAS X), at x10 and x16 objectives, respectively. The representative pictures for CD31 and Lyve-1 (Figs 3H to 3J and 4F to 4H) were extracted from these mosaics.

A homemade macro on the ImageJ software (https://imagej.nih.gov/ij/) was used to measure the lymphatic vessel density and the area of each vessel per field of view on the CD31 or Lyve-1 stained images.

Proliferating Ki67-positive cells were evaluated by counting Ki67 positive-cells in a random selection of 100 nuclei stained by DAPI, 2 images per sample.

### In vivo evaluation of perfused blood vessels

To evaluate tumor vascular perfusion and blood vessel density, mice received an injection of FITC-albumin (20 mg/ml, 2 mg/kg; Sigma) into the tail vein. After a circulation period of 1 hour, mice were humanely killed and the tumors were processed as described previously. The slices of nine to thirteen tumors per time points were stained with the endothelial cell marker CD31 (BD Pharminogen) as the primary antibody. Alexa Fluor 594 (Thermo Fisher Scientific) was used to show in red the endothelial cells. The total number of blood vessels (CD31 positive) and non-perfused vessels (CD31 positive and FITC-albumin negative) were counted per field of view (Leica M205FA microscope, objective x10, FOV: 1.5052 mm^2^).

### Histological analysis

**Fig 5:**
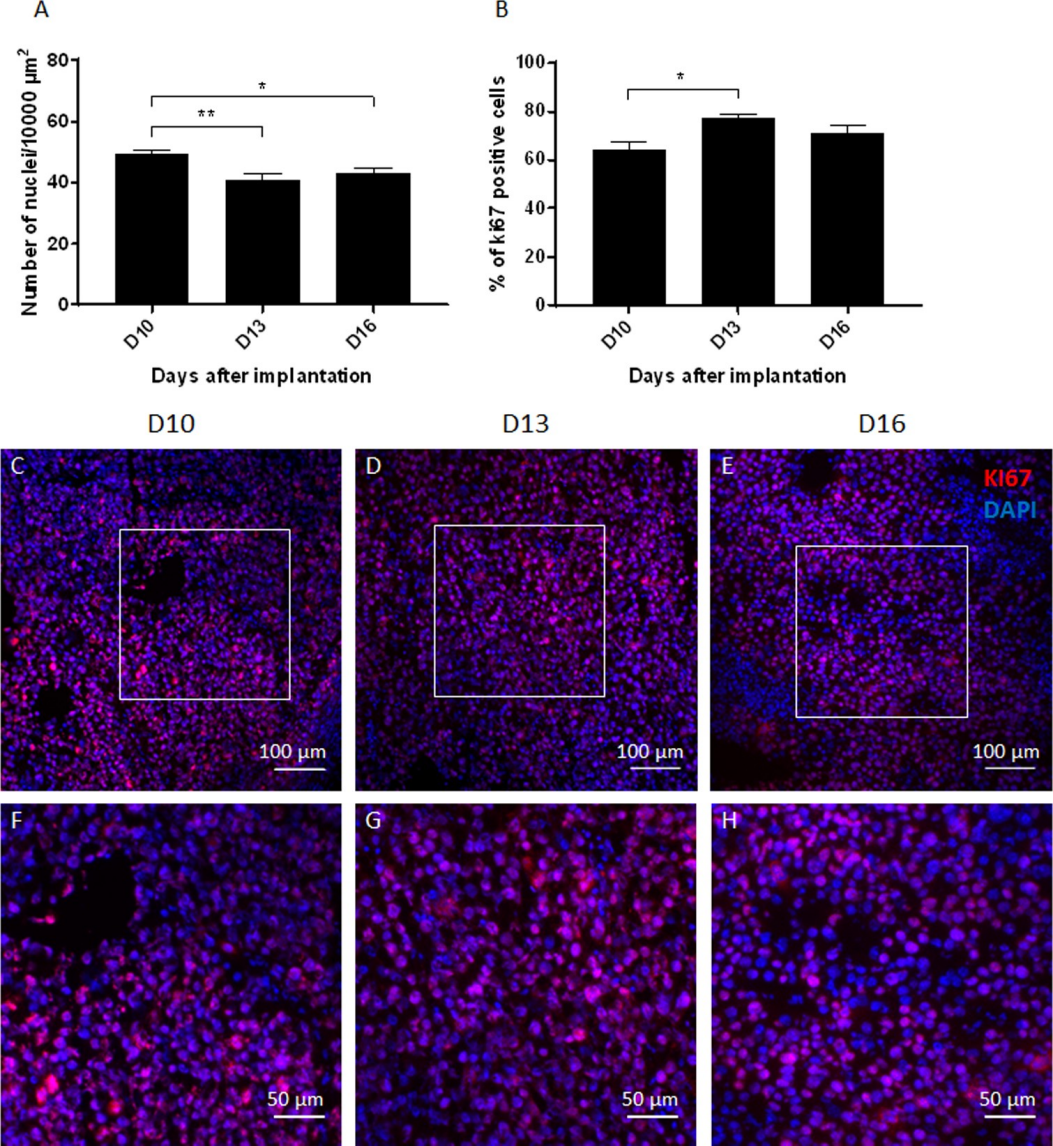
Quantification of tumor cell characteristics. (A) Time related changes in the cell density obtained by the number of nuclei in an area of 10000 μm^2^ of hematoxylin-eosin stained sections. (B) Time related changes in the percentage of KI67 positive cells among 100 Dapi-positive nuclei. (C, D, E) Representative merged images of KI67 and DAPI staining in frozen sections showing proliferating cells (Ki 67, red) among side blue Dapi-positive nuclei on D10 (C), D13 (D) and D16 (E) after implantation. (F, G, H) Zoom *in* of the white square in pictures A (F), B (G) and C (H) represented the cell density at higher magnification. Data shown as mean ± SEM. *: p<0.05, **: p<0.01. Scale bars: 100 μm (C, D, E) and 50 μm (F, G, H).

The tumor cell density was evaluated in hematoxylin and eosin (H&E) stained sections after melanin bleaching (Fig 5). Cryostat sections, 12 μm thick, were prepared from five tumors per time point. After fixation in cold methanol/acetone (v/v), the slices were bleached for 10 min in trichloroisocyanuric acid solution (TCCA, 10 g/L, Sigma) [20]. After different washing steps, the slices were stained with a modified H&E protocol. Briefly, after 2 quick baths in ethanol 100% and 1 in ethanol 95%, the slices were placed in hematoxylin for 10 min, washed quickly in distilled water and then kept under running water for 15 min, stained with eosin for 7 min, passed in ethanol baths at 75% and 95% (aqueous solutions), then into 100% ethanol. Slides were mounted with Eukitt mounting medium (Milan). Two images (Zeiss Imager M2 Light microscope with Cell D software microscope, objective x20, FOV 0.362 mm^2^) per sample were analyzed on Cell D. In each image, the number of nuclei in 10 squares per sample were counted manually within an overlayed grid (100 μm x100 μm).

### Protein extraction and Enzyme-linked immunosorbent assay (ELISA)

Seven pieces of different tumors per time points were cut, weighted and resuspended in an anti-protease buffer (5 mM NaOrVan, 10 mM NaF, 1 mM PMSF, 1X Mini Protease Inhibitor Cocktail, 300 μl/10 mg of tissue) for mechanical extraction of the proteins. The samples were shaken for 5 min (50 oscillations/sec), frozen (-20°C) and thawed (4°C) 3 times. Then, the samples were sonicated 2 times 30 seconds and centrifuged at 13500g for 20 min at 4°C. The supernatant was aliquoted and kept at -20°C after an assay of the protein content (Pierce BCA Protein Assay, ThermoFisher Scientific).

The concentration of VEGF-A in tumor tissue homogenates was determined by a sandwich enzyme-linked immunosorbent assay (ELISA, LSBio) in accordance with the manufacturer protocol. The samples were diluted 10 times with sample diluent provided by the kit and evaluated in duplicate.

### 3D visualization of the tumor vascularization: MicroangioCT

Four tumors at 13 days after the tumor cell implantation were anaesthetized as described above, heparinized, and then the thoracic aorta cannulated in the retrograde direction (Veflon i.v. catheter, 26 GA). After flushing out of the blood by an infusion of warm PBS containing heparin (20IU/kg bw), with the liver parenchyma cut in order to enable outflow, the mice were perfused with μAngiofil (perfusion speed 1 ml/min; total volume 3 ml) and then left undisturbed for 30 min at room temperature for the contrast medium to polymerize [21]. Afterwards, ears with tumor were fixed in 4% Paraformaldehyde PFA. Later the samples were scanned using a high-resolution desktop microCT, Skyscan-1272 (Bruker microCT, Kontich, Belgium) using the following parameters: isotropic voxel size 2.4 μm, 180 degrees scan, accelerating voltage 50 kV, no filter was applied. The obtained projections were used for a 3D-reconstruction using the NRecon Software Ver. 1.7.3.0 (Bruker microCT, Kontich, Belgium). The 3D-rendering of the obtained 3D-dataset was done using the CTvox Software Ver.3.3 (Bruker microCT, Kontich, Belgium).

### Preparation of single tumor cell suspensions

Seven to 8 tumors bearing mice per time point were used for this study at 10, 13 and 16 days after implantation; they were humanely killed and perfused with PBS. Following removal of the surrounding skin the tumors were gently removed and placed individually in collagenase solution (0,1% Collagenase IV, Sigma), cut up with fine scissors and incubated for 45 min at 37°C. The dissed tissue was first filtered using a Falcon Cell Strainers Mesh size 100 μm and 40 μm. The solution was then centrifuged at 450 g for 5 min at 4°C and supernatant discarded. To eliminate red blood cells the cell pellet was re-suspended in Lysis Buffer Solution (eBioscience), washed, centrifuged, re-suspended, and the residual cells counted in a Neubauer Chamber. Fc antibody fragment was blocked with a 1/100 solution of purified rat anti-mouse CD16/CD32 (Mouse BD Fc Block, eBioscience) in FC buffer (Flow Cytometry Buffer: PBS 1 X with 5% FBS and 2 mM Ethylenediaminetetraacetic acid (EDTA)). After incubation at 4°C for 30 min, samples were washed with FC buffer, centrifuged, and stained for membrane proteins with antibodies (described in the next section) by incubation at 4°C for 30 min. Finally, the cells were washed with FC Buffer, centrifuged and re-suspended in 500 μl of FC buffer. Propidium Iodine (PI, Thermofisher scientific) was added to identify viable cells. Flow cytometry studies were performed on a BD LSR II Special Order System (SORP) and subsequently analyzed was carried out with FlowJo V10 Software.

### Flow cytometry staining and analysis

**Fig 6:**
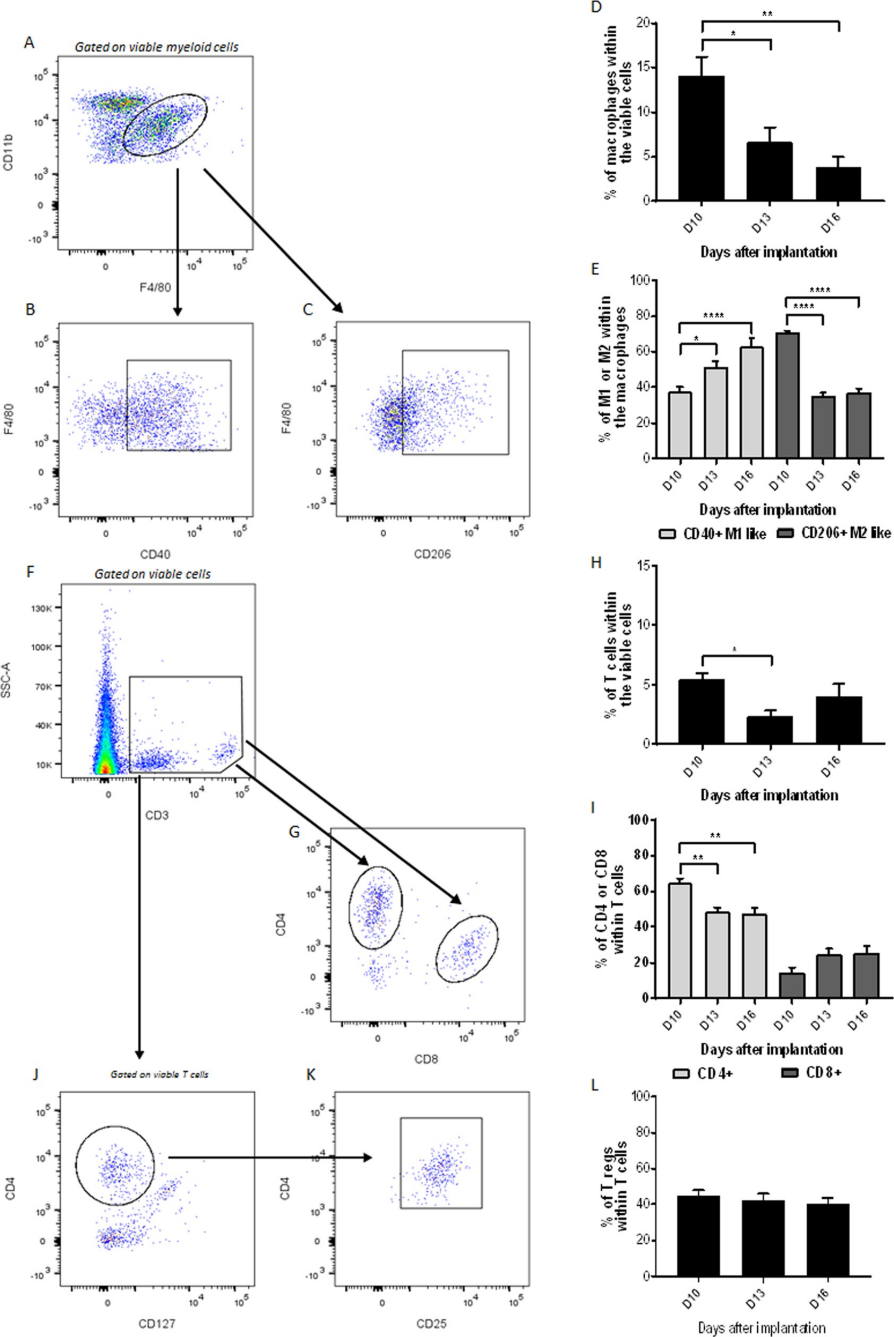
Characterization of macrophages and T cells. (A, B, C) Representative plots for the identification of the macrophage population (A) and of their two subtypes, M1-like (B) and M2-like (C) macrophages versus days after implantation. (D, E) Percentage of macrophages (D) and macrophage subtypes (E) versus days after implantation. (F, G) T cells (CD3) identification strategy (F) for the CD4+ and CD8+ subpopulations (G). (H, I) Percentage of T cells among viable cells (H) and T cells subpopulations (I) among all T cells, both versus days after implantation. (J, K, L) Representative plots for identifying T regulatory cells (CD4+CD127-, J, CD25+, K) among T cells, versus days after implantation. (L). Data shown as mean ± SEM. *: p<0.05, **: p<0.01, ***: p<0.001, ****: p<0.0001.

Macrophages were identified as being CD45+ (Biolegend, 103147), CD11b+ (Biolegend, 101245) or F4/80+ (Biolegend, 123149). The sub-populations of M1-like (CD40+, Biolegend, 124612) and M2-like cells (CD206+, Biolegend, 141723) were also differentiated. To identify T cells, CD3+ (Biolegend, 100349) cells were ‘gated’ and further set apart into two main T cells subtypes: CD4+ (Biolegend, 100555) and CD8+ (Biolegend, 100744). Natural T regulatory cells (Tregs) within the CD3+ T cells, were identified by their CD4+ CD127-reactivity (Biolegend, 135014). The Tregs identification was confirmed by their expression of the membrane protein CD25 (Biolegend, 102024). The gating parameters are described in Fig 6A, 6B, 6C, 6F, 6G, 6J and 6K.

### Number and volume of metastases

**Fig 7:**
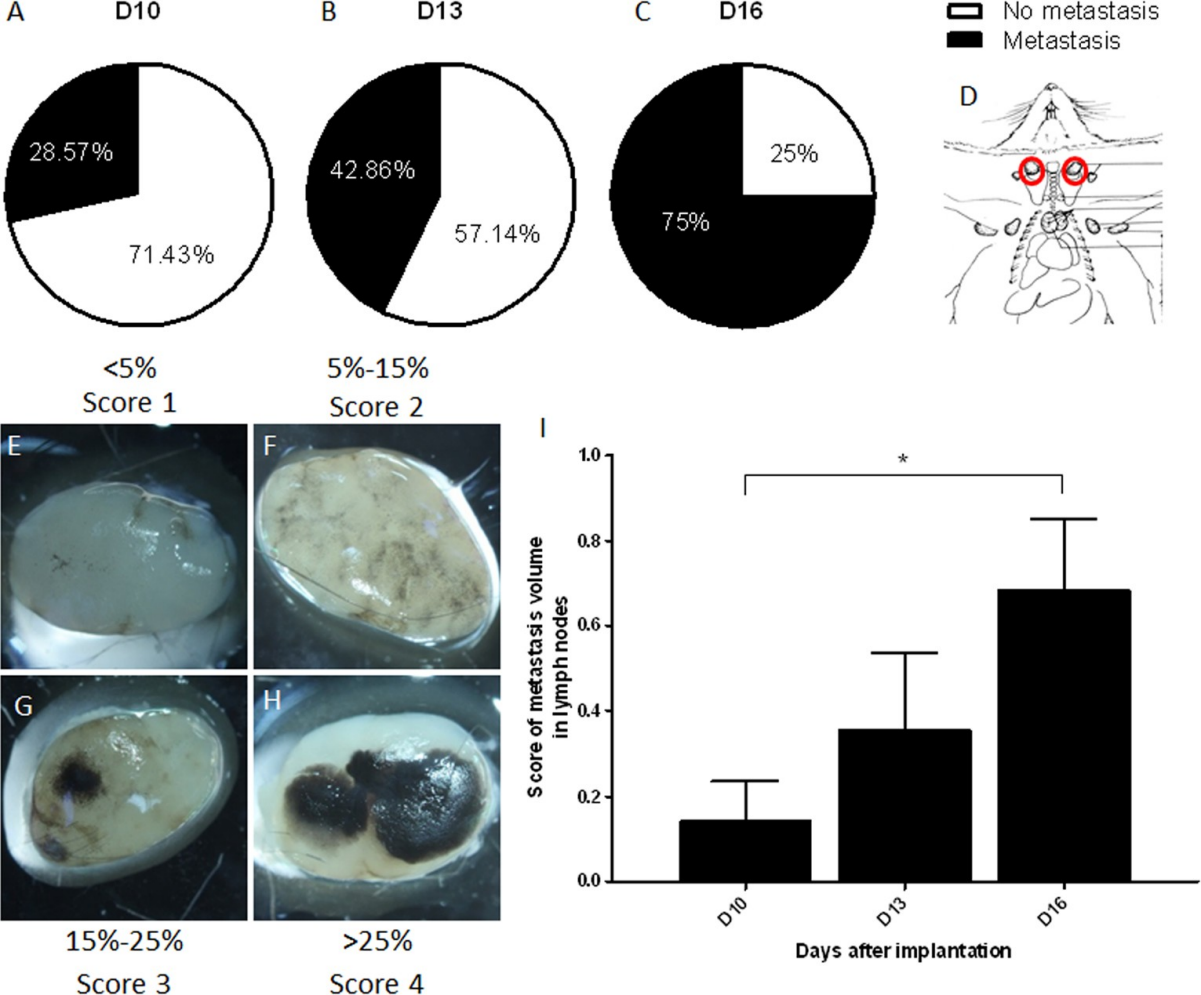
Metastases. (A, B, C) Percentage mice with melanotic cervical lymph node metastases on D10 (A), D13 (B) and D16 (C). (D) Localization of sampled lymph nodes (red). (E, F, G, H) Representative pictures of cervical lymph nodes harboring different metastatic volumes. Each node illustrates the amount of metastatic black tissue that characterizes the semi-quantitative scores of 1 to 4. (I) Estimation of the metastatic volume based on the score in E, F, G and H pictures versus days after implantation. Data shown as mean score ± SEM. *: p<0.05.

The two largest superficial cervical lymph nodes were selected from the 7–8 mice sampled at 10, 13, and 16 days after implantation. The lymph nodes were fixed in a 4% PFA solution and examined macroscopically. The percentage of mice bearing ear melanomas that showed cervical lymph node metastases, and the scoring system to evaluate the volume occupied by those metastases are presented in Fig 7E–7H. Briefly, score 1 was attributed to lymph nodes covered by less than 5% of metastasis, score 2 between 5% to 15%, score 3 between 15% to 25% and score 4 by more than 25% of metastasis.

### Statistical analyzes

GraphPad Prism (GraphPad Software, San-Diego, USA) one-way ANOVA (ANalysis Of VAriance) with Tukey post-test program have been used. Values were considered significantly different when p<0.05. The results are presented as mean values with a ± standard error of the mean (SEM). A nonparametric Spearman correlation test was used to evaluate the correlation between the percentage of black tumors and the size of the tumors (Fig 1G).

## Results

### The external aspect of the tumor (criteria for established tumors)

After surgery, the clots containing tumor cells were not visible through the skin (S2A Fig). About a week after implantation they had the appearance of a grayish bulb (Fig 1D and S2C and S2D Fig). At this stage, the growing masses have a low amelanotic cell density and a high leukocyte density (S2E to S2H Fig). Growing masses, which were turning black, were considered as established tumors (Figs 1C, 1E and S2D)

The number of established tumors increased significantly with time after implantation. Between D8 and D9 after implantation, the number of black tumors increased by a factor of 2.48 (20.7% ± 6.1% at D8 versus 51.3% ± 7.6% on D9, p<0.0001). Between D10 and D11, around 14% more tumors were classified as established (81.2% ± 10.9% at D10 versus 95.2% ± 2.3% at D11, p<0.05, Fig 1F). A significant correlation between the percentage of black tumors and their size was observed between D8 and D11 (Fig 1G, r = 1, p = 0.0417).

### B16-F10 melanoma in the ear pinnae–a rapid tumor growth

With a total of 135 tumors, and despite a slight heterogeneity within the growth rates, there were no significant differences between the 5 experimental replicates (p>0.05) (Fig 2A). The tumor size increased nearly exponentially from D11, when 95.2% of the tumors were considered as “established” *i*.*e*. became black (Fig 1F). The tumor volume doubling time was, between 1.73 days (experiment #3) and 1.99 days (experiment #2) (Fig 2B).

### Number and size of blood vessels

The blood vessel density decreased significantly between D10 (136.2 ± 83.5 blood vessels per FOV) and D16 (68.3 ± 10.6 blood vessels per FOV, p<0.01, Fig 3A and 3E–3J). However, the blood vessel area was not significantly modified (1002.1 μm^2^ ± 459.1 μm^2^ at D10 versus 1215.3 μm^2^ ± 557.4 μm^2^ at D16, p>0.05, Fig 3B). By D10, the percentage of perfused blood vessels was around 85% (83.6% ± 5.8%) and remained at that value (87.8% ± 5.9% on D13 and 84.3% ± 5.5% on D16, p>0.05, Fig 3C). The evaluation of VEGF-α production in tumors showed a significant increase between D10 (183.4 ± 78.2 pg/ml of protein) and D16 (406 ± 156.2 pg/ml of protein, p<0.01, Fig 3D.

3D-visualization of the tumor vasculature was carried out using microangioCT with μAngiofil as intravascular contrast agent (Fig 3K and 3J). The feeding vessels (blue arrows) entered the tumor mass from the base of the ear branching in smaller micro-vessels, but not all reached the superficial part of the tumor mass. The tumor vasculature was dense and chaotic (*e*.*g*., varying vessel diameters, irregular contours). There were areas with a dense network of smaller vessels (marked with asterisks), as well as many disproportionate bigger vessels (black arrows) (Fig 3J).

### Number and area of lymphatic vessels

The number of Lyve-1 immune-stained lymphatic vessels decreased significantly between D10 and D13 (from 28 ± 12 to 12 ± 6.5 lymphatic vessels per FOV, p<0.01, Fig 4A). The area occupied by lymphatic vessels, outlined by the external contour of Lyve-1 immuno-reactive endothelia, remained relatively constant with time (1211.3 μm^2^ ± 746.6 μm^2^ on D10, 1127.3 μm^2^ ± 461.7 μm^2^ on D13 and 939.2 μm^2^ ± 219.5 μm^2^ on D16, p>0.05, Fig 4B).

### Tumor cell density and proliferative index

The melanoma showed a significantly different cellular density between D10 (49.5 ± 2.6 cells/10,000 μm^2^) and D13 (40.9 ± 4.7 cells/10,000 μm^2^, p<0.01) and also between D10 and D16 (43.4 ± 3.1 cells/10000 μm^2^, p<0.05, Fig 5A). However, no significant differences were found between D13 and D16 (p>0.05). The observation of Ki67 immuno-staining indicated that the tumor mass was mainly composed of proliferating cells (>64.1%) (Fig 5B–5H). The percentage of these proliferating cells significantly increased between day 10 and day 13 (64.1% ± 8.3% at D10 versus 77.2% ± 4.5% at D13, p<0.05).

### Characterization of macrophages and T lymphocytes

The immune-phenotyping of cell suspensions taken from tumors, performed by flow cytometry, showed the presence of a significant macrophage population (14.1% ± 5.7% of viable cells) on D10. This had significantly decreased to 3.9% ± 3.2% on D16 (p<0.01, Fig 6D). However, the sub-population of M1-like macrophages (CD40+), among the macrophage population, increased significantly between 10 (37.7% ± 7.1%) and 16 days after implantation (62.8% ± 14.4%, p<0.0001), whereas the number of M2-like macrophages (CD206+) decreased significantly over the same period (70.8% ± 3% *versus* 37.3% ± 5.4% among macrophage population, p<0.0001, Fig 6E).

The proportion of T cells within viable tumor cells (5.5% ± 1.2% on D10) was significantly lower at D13 than at the other time points (2.3% ± 1.4% on D13 *versus* 4% ± 2.7% on D16, p<0.05, Fig 6H). Moreover, a significant decrease of CD4 positive T cells among the CD3 cell population was visible between D10 (64.6% ± 7.1%) and D16 (47.7% ± 7.8%, p<0.01, Fig 6I). CD8 positive T cells increased between D10 (14.6% ± 7.2% of CD3+ cells) and D16 (25.5% ± 10.1% of CD3+ cells, p>0.05, Fig 6I). The sub-population of natural T regulatory cells (CD127 negative amongst the CD3 cell population) remained constant over the period from D10 (44.9% ± 8%) to D16 (40.1% ± 10%, p<0.05, Fig 6L).

### High frequency of regional lymph node metastases

On day 10, metastases were found to be present in regional cervical lymph nodes of 28.6% of mice bearing ear melanomas. This percentage increased to 75% of mice with ear melanomas on D16 (p<0.05, Fig 7A and 7C). Using the arbitrary score system defined in Fig 7E–7H, it appeared that the metastatic volume also increased significantly between D10 and D16 (0.14 ± 0.24 *versus* 0.68 ± 0.46 respectively, p<0.05, Fig 7I). Metastases were not detected in the lungs or liver at these time points.

## Discussion

In this study, a B16-F10 mouse melanoma model is described as a primary tumor, locally implanted in the ear pinnae of C57BL/6J mice. This model showed highly reproducible results concerning tumor development tumor growth among 5 repeated studies. The tumor was highly vascularized, with a near-exponential growth pattern and high proliferative rates. Both macrophages and T cells were present in the tumor, making it a relevant model for studies involving immune responses. The intra-tumoral macrophage population decreased with time, with a switch in the proportion of M1-like *versus* M2-like macrophages. The proportion of Cd4+ T cells decreased in the quasi-constant population of T cells, but the number of CD8+ T cells and regulatory T cells remained constant. The tumor implantation also allowed the evaluation of the spontaneous metastatic development of B16-F10 tumors into local cervical lymph nodes without the risk of the artificial seeding of tumor cells into the lymphatic vessels associated with injection pressure [19].

The published literature shows that the B16 melanoma is commonly injected into the flank skin, into the footpad or into the tail vein for the F1, BL6 and F10 cell lines, respectively [13,14]. In the present study, B16-F10 cells are transplanted into the ear mixed in a compact, easily handled, clot composed of fibrinogen, thrombin and calcium. Unlike subcutaneous injections of tumor cells into the ear, the removal of a small piece of ear cartilage allowed the tumor to grow on both sides of the ear pinnae [17]. The replacement of cartilage by a tumor matrix can be used to improve the evaluation of the angiogenic potential of the tumor since the tumor grows in previously unoccupied space, within the avascular zone. Therefore, by default, all non-fibrin structures are new, induced by the tumor growth and the healing processes. The simplicity of the distinction between pre-existing and induced blood and lymphatic vessels, but also matrix components, is an essential value of this model.

The tumors are accessible and can also be followed effortlessly. Simple tools, such as a short anesthesia, a binocular microscope and calipers suffice to measure tumor growth, without error due to a non-accessible area such as in the internal organs. Moreover, the tumor measurement in the ear can be performed in 3D (on length, width and thickness). The location on the surface of the ear allows easy access for *in vivo* imaging with a fluorescent stereo-microscope (S3 Fig) or with a two-photon microscope [18,22]. Transgenic melanoma cells or *in vivo* immuno-stained macrophages and lymphocytes may also be imaged, as well as blood vessel alteration after anti-angiogenic treatment.

In the literature it has been reported [23,24] that tumors, up to the 1–2 mm^3^, can persist for years without induction of angiogenesis. Those tumors are considered as “dormant” and obtain the oxygen and nutrients by simple diffusion. Rapid growth and expansion start after the release of angiogenic factors (mainly VEGF) and the recruitment of endothelial cells from the surrounding stroma *i*.*e*. tumors turn to an angiogenic phenotype [24]. In the present study, the implantation of a total volume of 10 μl (thrombin, fibrinogen and melanoma cells), creates a solid clot of around 10 mm^3^. Thus, tumor size alone with this method cannot be used as a reliable criterion to determine if a tumor has established itself and will expand. It was found that tumor opacity was a very consistent sign; as soon as the tumor became visible (turned to grey), they started to grow continuously. The very tight correlation between tumor opacity and tumor volume prompted the use of melanoma opacity as a criterion for the establishment of a B16-F10 tumor. The “established tumors” in the present model demonstrated almost identical growing curves and aggressiveness, which is essential for evaluation of different treatment modalities by choosing animals at the same stage of tumor development.

In the first week after implantation, the tumor clots were not visible and only after 7–8 days after implantation a grey bulb appeared in the ear (Fig 1E). Histological observations (S2E to S2H Fig) showed that the growing mass contained great number of leukocytes. The proliferating melanoma cells were showed as an accumulation of melanosomes, even though only a few cells expressed melanin. It is suggested that most of the melanosomes in the proliferative phase are amelanotic, *i*.*e*. in stage I and II. In a study of Danciu *et al*. (2015), the authors also have observed an increase of melanin in B16-F10 melanoma tumor subcutaneously injected into the flank [25].

With the onset of tumor-angiogenesis, established tumors are able to expand into healthy tissue and metastasize to other organs [26]. The high cell density and the high proliferation index observed in B16-F10 tumors are related to the fast tumor growth between D10 and D16 (increased by 8 times) after implantation. In parallel, a slight decrease of the blood vessel density was observed (by a factor of 2 between D10 and D16).

The discrepancy between the tumor size and vascular density is due to the high proliferative activity of melanoma cells and rapid augmentation of the tumor. Up to day 10, the proliferative ratio is high but the number of melanoma cells still small and the tumors are expanding in a modest manner. This allowed adequate vascularization. Due to the exponential tumor growth and in spite of the neoangiogenesis, the relative number of the blood vessels become smaller and this resulted in reduced vascular density. Those results are in accordance with data observed in gliomas, another type of rapid growing tumor [27].Despite this decrease, it is suggested that new vessels have still been created by angiogenesis. In opposite to the thinly and hierarchical organized microvessels of the normal ear tissue, the capillaries of the growing melanomas are tortuous, enlarged with a high density (Fig 3K, 3J and 3L). It is obvious that the tumors did not adopt the already existing normal vessels of the ear and induced neo-angiogenesis to support the rapid tumor growth. In accordance with this statement is the increasing amount of VEGF-α from D10 to D16 (Fig 3D). Among the tumor blood vessels, a constant 15% of non-perfused vessels was observed. The sprouting angiogenesis, induces the creation of new blood vessels from pre-existing vessels, with the protrusion of endothelial cell extension led by tip cells [28]. The vascular structure resulting from sprouting angiogenesis in tumor is chaotic, with leaky endothelial cells, multiple fenestration and poor pericyte/smooth muscle cells coverage. Such blood vessels have a dysfunctional ability of the endothelium to transport fluid and limit the perfusion of the tumor [29–32]. Such non-functional vessels could explain the presence of un-perfused vessels in the present model.

Macrophages and lymphocytes are two significant players in the tumor immune response. Surprisingly, a switch of the macrophage subtypes was seen between day 10 to 16 post implantation in these B16-F10 tumors (Fig 6E). The M1-like macrophages, known as pro-inflammatory macrophages [33], showed an increased infiltration. This could be explained by the fast tumor growth that induces intra-tumoral pressure and distortion of the surrounding skin leading to peripheral inflammation. M2-like macrophages, defined as pro-angiogenetic and pro-tumor macrophages [33], decreased in time. This phenomenon could be an indication of the promotion of tumor development and angiogenesis in the earlier stages, necessary for tumor expansion [34]. It is well-known that the anti-tumor immune responses (including T cells effectiveness) are influenced by an unfavorable tumor microenvironment. The latter, together with a very rapid and aggressive tumor growth, observed in the present study, could be one of the main reasons of the decrease of the CD4+ T cells as a sign of the cancer evading immune-surveillance. Moreover, a decrease of CD4+ T cells could be associated with unfavorable outcomes since an absolute increase of CD4+ and CD8+ cells, also after therapies, is associated with a favorable prognosis in patients [35]. On the other hand, in the present tumor model, it seems that the cytotoxic reaction directly executed by CD8+ T cells is kept constant.

Regulatory T cells (Tregs) in tumors are behaving as mediators of the immune response. It is known that they carry out immunosuppressive functions on the other tumor infiltrating immune cells. Nevertheless, the correlation of these properties to the development and outcomes of the different malignancies is still controversial [36]. In the presented melanoma model, it has been possible to underline a constant and abundant Tregs population suggesting that there is an active work of mediation of the immune response at the different stages.

The B16-F10 melanoma cells, injected in the tail vein, are commonly used to study the development of lung metastases [13,14]. In the present model, no lung or liver metastases were observed in the mice bearing ear melanomas. However, metastases developed in cervical lymph nodes in 75% of mice 16 days after implantation (Fig 7C). Metastasis development in regional lymph nodes is a frequent and early event in melanomas, since a biopsy of sentinel lymph nodes is perform to establish a diagnosis of the melanoma stage [37]. However, the latest observation time point in the present study, 16 days after implantation, was probably too early for the detection of metastases development in distant organs like in lungs, where they appear around 3 weeks after tumor inoculation [38]. In human patients bearing cutaneous malignant melanomas, lymphatic micro-vessel density is associated with the presence of regional lymph node metastases and shorter patient survival. The density of such tumor-associated lymphatic networks has been proposed as tool for selecting high-risk patients for new anti-melanoma therapies [39].

## Conclusion

A locally transplanted B16-F10 melanoma model has been established and characterized. It is a highly reproducible model which is easy to follow. The localization of the tumor in the ear permits, for instance, testing new methods of radiotherapy as vital organs are not exposed. Basic characteristics of the lymphatic vessels, tumor and immune cells, and of the metastatic potential of ear melanomas in this study may be of practical value when using this model for experimental investigations of melanomas.

## Supporting information

S1 Fig Surgical steps for tumor implantation. (A) Scalpel incision though the skin on three sides of a square (5x5x5 mm) on the ventral side of the ear (black line). (B) Reflected flap of the skin to expose the ear cartilage (black dashed lines). (C) Four shorter full thickness incisions in the ear cartilage by (4 mm^2^, grey dashed lines)) (D) Removing of part of the cartilage square (white area). (E) Implantation of the clot, with tumor cells into the square vacated by the removal of cartilage (grey circle). (F) Closing of the skin flap with surgical glue along the line of the skin incisions (black lines).
(TIF)

S2 Fig Tumor evolution within the 1^st^ week after implantation. (A) Picture of the mouse ear 2 dpi, the tumor clot is not visible anymore. (B) Picture of the mouse ear 4 dpi, the implantation site becomes pinkish. (C) Picture of the mouse ear 6 dpi, the tumor mass becomes visible. (D) Picture of the mouse ear 8 dpi, a non-established melanoma is visible. (E, F) Representative image of non-established tumor, 6 dpi. (H&E staining, x 20). (G, H) Representative image of non-established tumor, 8 dpi (H&E staining, x 20).
(TIF)

S3 Fig *In vivo* staining feasibility. *In vivo* staining and imaging of blood vessels (red) and nucleus (blue) in B16-F10 implanted tumor.
(WMV)

S1 Material and method (DOCX)
