# Supplementary figures and images for: Characterization of a B16-F10 melanoma model locally implanted into the ear pinnae of C57BL/6 mice
Source: PLoS One. 2018 Nov 5;13(11):e0206693. doi: 10.1371/journal.pone.0206693 (PMC6218054; doi:10.1371/journal.pone.0206693)

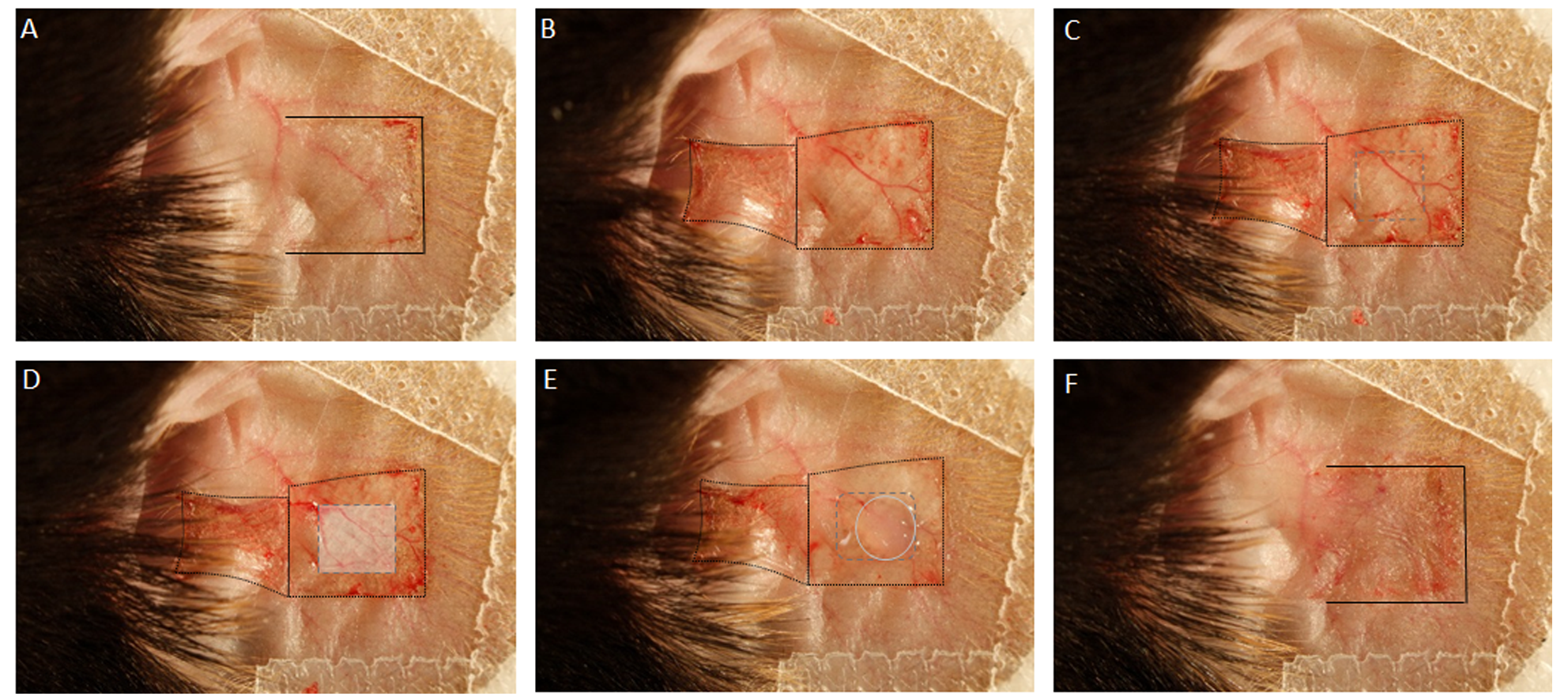

Supplement: S1 Fig — (A) Scalpel incision though the skin on three sides of a square (5x5x5 mm) on the ventral side of the ear (black line). (B) Reflected flap of the skin to expose the ear cartilage (black dashed lines). (C) Four shorter full thickness incisions in the ear cartilage by (4 mm2, grey dashed lines)) (D) Removing of part of the cartilage square (white area). (E) Implantation of the clot, with tumor cells into the square vacated by the removal of cartilage (grey circle). (F) Closing of the skin flap with surgical glue along the line of the skin incisions (black lines). (TIF) [file pone.0206693.s001.tif]

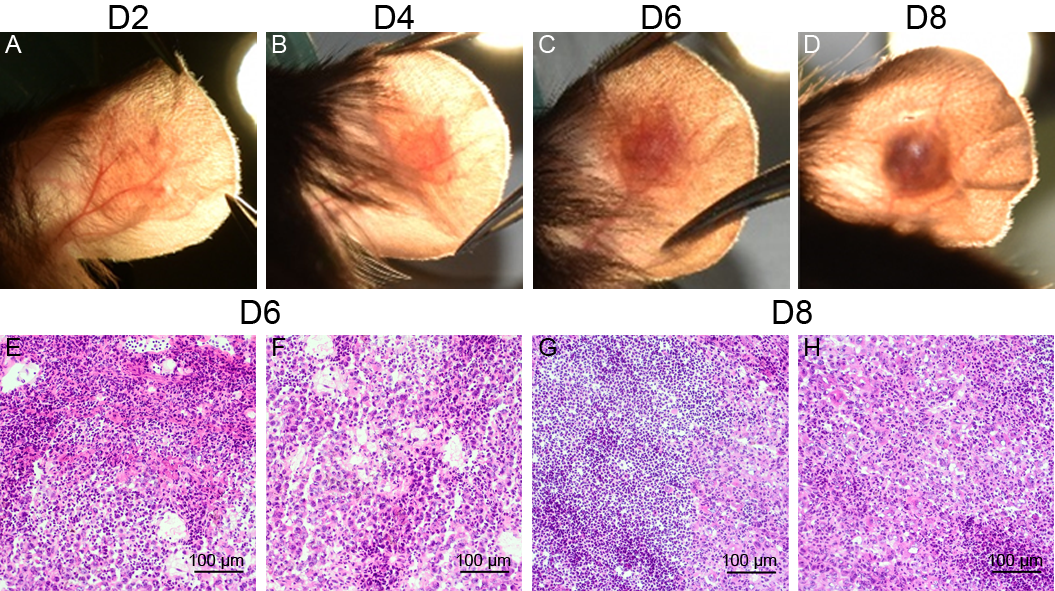

Supplement: S2 Fig — (A) Picture of the mouse ear 2 dpi, the tumor clot is not visible anymore. (B) Picture of the mouse ear 4 dpi, the implantation site becomes pinkish. (C) Picture of the mouse ear 6 dpi, the tumor mass becomes visible. (D) Picture of the mouse ear 8 dpi, a non-established melanoma is visible. (E, F) Representative image of non-established tumor, 6 dpi. (H&E staining, x 20). (G, H) Representative image of non-established tumor, 8 dpi (H&E staining, x 20). (TIF) [file pone.0206693.s002.tif]
