# Supplementary material for: Characterization of a B16-F10 melanoma model locally implanted into the ear pinnae of C57BL/6 mice
Source: PLoS One. 2018 Nov 5;13(11):e0206693. doi: 10.1371/journal.pone.0206693 (PMC6218054; doi:10.1371/journal.pone.0206693)
Supplement: S1 Material and method — (DOCX) [file pone.0206693.s004.docx]

**Supporting information**

**Protocol for *in vivo* staining and imaging**

The tumor was stained and imaged *in vivo* as described in Guç *et al*., 2014 [18]. Briefly, after anaesthesia, the mouse was placed on the back and the ear was attached to a glass stack with surgical glue. The ventral skin of the ear was cut with a scalpel along the antihelix of the mouse pinna, and the skin was gently peeled of with tweezers. The open tumor was incubated with Ringer's buffer complemented by 100 μl of thrombin (5 U/ml, Sigma) for 5 min to stop the bleeding of small blood vessels and then washed 2 times with normal Ringer’s buffer. Primary antibody CD31 (1/100, BD Pharminogen) was applied for 15 min in Ringer’s buffer supplemented by donkey serum (10%, Sigma) and aprotinin (2.5 mg/ml, Sigma). After washing 2 times the tumor with normal Ringer’s buffer, the tumor was incubated with the secondary antibody (Alexa Fluor 594, 1/200, Life Technology) in Ringer’s buffer supplemented by donkey serum (10%, Sigma), aprotinin (2.5 mg/ml, Sigma) and Dapi (1 µg/ml, Sigma). After washing, a freshly prepared Ringer's buffer containing sodium ascorbate (140 mM), HEPES (10 mM), KCl (4 mM) and CaCl2 (5 mM), at a pH of 7.5 was applied and a coverslip was deposited on the tumor before to start imaging. The movie was performed on a Leica M205FA stereomicroscope with Leica software LAS X.
